# Midwifery models of care in the context of increasing caesarean delivery rates

**DOI:** 10.2471/BLT.24.293035

**Published:** 2025-05-12

**Authors:** Tanya Doherty, Sheila Clow, Margreet Wibbelink, Mariatha Yazbek, Soo Downe

**Affiliations:** a Health Systems Research Unit, South African Medical Research Council, Francie van Zijl Drive, Parow, 7501 Cape Town, South Africa.; b Division of Nursing and Midwifery, University of Cape Town, Cape Town, South Africa.; c Department of Nursing Science, Nelson Mandela University, Nelson Mandela Bay, South Africa.; d Department of Nursing Science, University of Pretoria, Pretoria, South Africa.; e School of Nursing and Midwifery, University of Central Lancashire, Preston, England.

Clinicians and researchers have used the concepts of too little too late, and too much too soon for almost a decade to describe disparities in access to and levels of use of clinical procedures in maternity care worldwide.^1^ The case of caesarean delivery is the most widely debated of these procedures. In many countries, rates are below safe levels in particular geographies or population groups, indicating that mothers and babies may be experiencing adverse outcomes due to a lack of access to the operation. On the other hand, the World Health Organization (WHO) has stated that, while low caesarean delivery rates indicate poor coverage of essential maternity care, no public health benefit exists when the rate exceeds 10–15% at a population level.

Most women would prefer physiological labour and birth if this is safe and well supported.^2^^,^^3^ Performing medically unnecessary caesarean deliveries undermines women’s choices and leads to a rise in adverse outcomes for women and newborns and generates significant extra costs for health systems.^4^ Projections estimate that, by 2030, about one third of all newborns will be born surgically. Of the five countries with the highest caesarean delivery rates globally (Brazil, Cyprus, Dominican Republic, Egypt and Türkiye), four are middle-income countries and one is high-income.^5^ In some middle-income countries, both very low and very high rates are problematic. For instance, in 2020, in the southern state of Telangana in India the overall rate was 7.5 times higher than in the north-eastern Meghalaya state.^6^ Predictors of caesarean delivery included higher education, delivering in a private hospital and high socioeconomic status. Data at national levels on caesarean delivery rates within public and privately funded health sectors are scarce due to the differences in health information systems and clinical governance oversight between the two sectors. An exception is Australia, where national-level monitoring data are available showing a caesarean delivery rate of close to half among women attending private hospitals and one third among women attending public hospitals in 2022.^7^ Differences between the public and private sectors are also found in a middle-income country, South Africa, where caesarean delivery rates are 32% (284 459 caesarean deliveries out of 883 244 live births) in the public sector;^8^ while in the private sector, caesarean delivery rates of 77% (81 103 caesarean deliveries out of 105 485 live births)^9^ are among the highest in the world.

The drivers of rising caesarean delivery rates are multifaceted and include factors relating to women, society, health workers, financing arrangements and health-care organizations. Underlying these rising caesarean delivery rates is the lack of investment, commitment and advocacy for midwifery models of care. The midwifery philosophy of care promotes a person-centred approach to care; values the women–midwife relationship and partnership; optimizes physiological, biological, psychological, social and cultural processes; and uses interventions only when indicated.^10^ A recent systematic review reveals that approaches such as midwife-led continuity of care models result in higher rates of normal physiological vaginal births, reduced caesarean sections, safer outcomes, more positive birth experiences and lower costs.^11^ In October 2024, WHO published a global position paper on transitioning to midwifery models of care.^10^

In middle-income countries such as Brazil, Egypt, South Africa and Türkiye, with national or subgroup caesarean delivery rates exceeding 50%, considerations exist for transitioning to midwifery models of care. In such contexts with high rates of medically unnecessary caesarean deliveries, the autonomy of midwives has been eroded within maternity care teams, and both midwives and obstetricians have progressively lost skills in managing physiological labour and birth and in conducting assisted vaginal birth. Midwives in these contexts spend less time supporting and enabling women in labour and more time as assistants in operating theatres. Shifts towards midwifery models of care therefore need to include comprehensive, team-based pre- and in-service skills training packages to rebuild confidence and competence in midwifery practice. Programmes also need to include education in the value of, and skills in watchful attendance. The term expresses a combination of continuous support, clinical assessment and responsiveness, and requires a high level of expertise that enables a calm and confident space in which labour and birth can unfold spontaneously, while also being acutely alert to emerging signs that the woman wants and/or needs more active support or referral.

The differences in power and authority between health professionals in maternity teams, especially in traditional medically oriented maternity services, are important barriers to shifting to midwifery models of care. A focus on improving professional trust, communication and collaboration within and across maternity teams including midwives, obstetricians, anaesthetists, nurses and paediatricians, needs to support the move towards midwifery models of care.^3^ Engagement and collaboration between professional societies at global and national levels are important components of garnering interprofessional support for shifts to midwifery models of care and for supporting national policy-makers with guidance. The International Confederation of Midwives has published a policy brief on implementing midwife-led birth centres.^12^ This brief outlines key considerations for implementing this model, including supportive national leadership and governance; effective and sustainable financing; high-quality midwifery care that is valued by communities; and interdisciplinary and interfacility trust and collaboration. Engagement with the International Federation of Gynaecology and Obstetrics is also critical to encourage recognition of complementary professional strengths and to dissipate fears of competition.

In 2021, the State of the World’s Midwifery report estimated a global shortfall of 900 000 midwives. Addressing this gap will require scaling up high-quality direct-entry midwifery training. Programmes that include midwifery training within general nursing courses are lengthy, often condense the midwifery curriculum, contribute to high attrition among midwifery-qualified personnel and are unlikely to meet the current demand for midwives, particularly those who meet the International Confederation of Midwives’ essential competencies for practice. In addition to increasing the training of midwives, decision-makers at government level should consider fair and appropriate remuneration that is commensurate with the level of skill, time commitment and responsibility required of midwives. Doing so is particularly relevant because midwives are paid less than average among highly skilled workers in 39 out of 49 countries.^13^ The WHO position paper includes evidence that midwifery models of care increase job satisfaction and foster professional growth among midwives,^10^ which is a critical component of reducing midwife attrition and staff turnover.

Considering what types of organizational structures would facilitate transitions to midwifery models of care is critical. One example is recognized onsite midwife-led birth units that are distinct from obstetric units, and that enable triage on antenatal booking and ongoing assessment as to the appropriate level of care throughout pregnancy, labour and the postpartum continuum.^14^ Midwife units need to become mainstream, recognized clinical entities that could be located within the grounds of hospitals or in communities, considering context and geography.^14^

Shifting to midwifery models of care in settings with high rates of caesarean birth will also require large-scale, innovative behaviour change communication at a societal level. Communication should focus on restoring confidence in midwives as autonomous practitioners, restoring women’s confidence in their ability to give birth, reducing fears of spontaneous labour and vaginal births, and providing evidence-based, accessible, attractively presented information about the risks and benefits of different labour and birth options for women and newborns, including data on the longer-term life-course consequences. The critical need for such communication to start chronologically with the introduction or expansion of midwifery models of care should not be underestimated.

To avoid continuing health system-related harm to many women and their neonates, middle-income countries must address their alarming rates of caesarean births by shifting to more person-centred, team-based midwifery models of care. In a case study of midwife-led birth centres in four low-middle income countries (Bangladesh, Pakistan, South Africa and Uganda), four universal themes emerged that described the enabling factors influencing success of such centres: (i) an effective financing model; (ii) quality midwifery care that is recognized by the community; (iii) interdisciplinary and interfacility collaboration, coordination and functional referral systems; and (iv) supportive and enabling leadership and governance at all levels.^3^ Several countries provide examples of midwifery models of care that provide useful lessons to guide policy-makers in this health system transition (Box 1). The next steps required of health policy-makers to shift to maternity models of care are outlined in Box 2. The considerations raised in this article are critical to ensure the success of such shifts, valuing the autonomy, skills and potential of midwives to transform the pregnancy, birth and postnatal care experiences for women and families, for their short and longer-term benefit.

## Box 1 Examples of middle-income countries that have implemented midwifery models of care

### *Brazil*

Brazil is an example of a country that has seen large increases in caesarean births, reaching more than half in 2021 in the public sector while exceeding 88% in the private sector (4188 caesarean deliveries out of 4765 births in a national hospital-based sample). In response to rising caesarean delivery rates, the Brazilian government implemented freestanding midwifery units in 1998 and shortly after, alongside maternity units were introduced, situated within a hospital providing midwife-led continuity of care with obstetrician and neonatologist support when needed. These models of care exist only in the public sector, are fully funded by the State and situated mainly in urban areas. Having a mix of organizational structures, both onsite and freestanding, allows for greater flexibility in birth care management within this context. In addition to the establishment of these birthing units, the Brazilian government recognized the value of nurses and midwives in promoting safe midwifery care, offered financial incentives to train more midwives and developed strategies for professional inclusion and recognition. These incentives included investing in the qualification of midwives through direct-entry midwifery training, and promoting continuous education for all professionals providing maternal and perinatal health care.

### *South Africa*

In South Africa, free-standing midwife obstetric units were established in the 1970s. These units, with linkage to a secondary hospital and through midwives, provide antenatal and intrapartum care to low-risk women. In some provinces, additional models of onsite midwife-led birth units have been established in the grounds of hospitals for ease of referral in the event of complications. As with Brazil, these units are only available in the public sector and are fully State funded. They are fully integrated into the primary-care system and have strong clinical governance. Monthly perinatal review meetings are conducted at the midwife obstetric units and representatives from these units are required to attend the monthly perinatal review meetings at the referral hospital. A few private freestanding maternity units exist for out-of-pocket or privately insured women, but these are rare and struggle with sustainability concerns. South Africa is currently in the process of planning a national health insurance scheme where the scheme will be the strategic purchaser of health-care services from both the public and private sectors. Midwife-led birthing centres would lend themselves to public–private engagement to increase access for women to high-quality midwifery models of care.

## Box 2 Next steps required of policy-makers to shift to midwifery models of care^a^

### Commit and invest

- Promote supportive and enabling leadership and governance at all levels of a unified national strategy on scaling up midwifery models of care.
- Establish a national system for monitoring progress towards scaling up midwifery models of care.
- Ensure national monitoring systems include caesarean delivery rates in public and private sectors.
- Design an effective, sustainable financing model that prioritizes domestic resources.
- Review the remuneration of midwives, commensurate with their level of skills and responsibility to promote a rewarding work environment.

### Educate, deploy and retain

- Consider establishing direct-entry midwifery training for countries without this option.
- Offer comprehensive, team-based pre- and in-service skills training packages to rebuild confidence and competence in midwifery practice.
- Promote interdisciplinary and interfacility collaboration, coordination and functional referral systems.

### Advocate and empower

- Implement innovative large-scale behaviour change communication to restore community confidence and trust in midwifery models of care.
- Strengthen interprofessional trust, respect and collaboration within and across maternity teams.
- Engage with national professional societies of midwives and obstetricians to co-design and reach agreement on the preferred operating model of team-based maternity care.
- Give voice to women’s experience of and preferences for care.

^a^ Arranged according to the three pillars of the Midwifery Accelerator.
